# Vision Sensor Based Fuzzy System for Intelligent Vehicles

**DOI:** 10.3390/s19040855

**Published:** 2019-02-19

**Authors:** Kwangsoo Kim, Yangho Kim, Sooyeong Kwak

**Affiliations:** 1 Department of Electronics and Control Engineering, Hanbat National University, Daejeon 34158, Korea; kskim@hanbat.ac.kr; 2 System Software Development Team, GLOBALSYSTEMS Co.Ltd, Daejeon 34104, Korea; kimyh@globalsystem.co.kr

**Keywords:** pedestrian protection, fuzzy system, behavior prediction, intelligent vehicle, movement analysis

## Abstract

Those in the automotive industry and many researchers have become interested in the development of pedestrian protection systems in recent years. In particular, vision-based methods for predicting pedestrian intentions are now being actively studied to improve the performance of pedestrian protection systems. In this paper, we propose a vision-based system that can detect pedestrians using an on-dash camera in the car, and can then analyze their movements to determine the probability of collision. Information about pedestrians, including position, distance, movement direction, and magnitude are extracted using computer vision technologies and, using this information, a fuzzy rule-based system makes a judgement on the pedestrian’s risk level. To verify the function of the proposed system, we built several test datasets, collected by ourselves, in high-density regions where vehicles and pedestrians mix closely. The true positive rate of the experimental results was about 86%, which shows the validity of the proposed system.

## 1. Introduction

It is known that vehicles traveling at high speeds are prone to accidents that can lead to serious injuries or even death of pedestrians, as well as of passengers and drivers. According to the AAA Foundation’s Traffic Safety Culture Index (TSI) survey, however, traffic accidents that are caused by vehicles traveling at much lower driving speeds on American urban and residential streets are also major problems [1]. In Korea, more than 65% of pedestrian casualties from car accidents occur on local streets in residential areas [2], where the sidewalks are not divided clearly from the motorways by a curb. In this environment, vehicles and pedestrians frequently mix up, and it is highly probable that pedestrians will be hit by vehicles. The low ability of children and elderly pedestrians to react to urgent situations is reported to be the main cause of accidents on local streets. Careless drivers who are engrossed in the other activities while driving (e.g., text-messaging, talking with passengers, taking a phone call, manipulating a car navigation device, etc.) are also blamed as another cause for such accidents [3,4].

The technology by which car accidents and passenger casualties could be avoided or diminished by utilizing electric, electronic, and control technologies, along with conventional mechanical technologies, are collectively referred to in the vehicle industry as vehicle active safety technology. Compared with the safety issues of protecting drivers and passengers, the pedestrian safety issue has been relatively recently imposed. In particular, the obligation to add pedestrian protection functions to vehicles is being pushed by the governments of Europe and America. The major car manufacturers are trying to cope with the changing environment by adopting or developing a variety of related technologies. Among the technologies necessary for implementing pedestrian protection features, computer vision technology is known to be the most prominent. This kind of active protection technology aims to detect pedestrians in front of vehicles and to avoid accidents by itself, as much as possible. Pedestrian detection technologies have been researched mainly in the intelligent surveillance and security industries, but, recently, thanks to the issues of pedestrian safety, the automotive industry has become very interested in commercializing this technology.

The product called City Safety (by Volvo, Gothenburg, Sweden), is the first commercialized product that gives visual and auditory warning signals when it detects any pedestrian in front of the vehicle, and even makes the vehicle stop when it perceives any danger of hitting pedestrians [5]. This system uses an on-board dedicated camera installed on the windshield, and several distance ultrasonic and LIDAR distance sensors. The Pedestrian Collision Warning (PCW) system by Mobileye is another camera-based commercial product that issues an alert by providing visual and audio warning before imminent collision with a pedestrian or cyclist [6]. 

These commercialized products simply use information on the distance from the vehicle to the detected pedestrians. The systems do not consider whether pedestrians are about to cross the road or have stopped on the side of the road. However, it can easily be inferred easily that the risk level of crashes can be very different depending on the movement of pedestrians. Pedestrians who are moving toward the vehicles may have a higher risk of collision than those who are standing beside the road or moving away from the vehicle or a road, even though their instantaneous positions are the same when detected. If the concept of pedestrian intention is added, a pedestrian safety system can be elaborated. Research has already been conducted to estimate pedestrian intention using three different techniques: trajectory-based approaches, motion-feature-based approaches, and skeleton-based approaches.

The first approach for analyzing pedestrian intention predicts pedestrian trajectories based on their recent track histories, and estimates their destinations [7]. This method is appropriate for crossroads with crosswalks. The 3D mapping technique proposed by Mogelmose et al. is another approach based on a trajectory, which maps the pedestrians’ trajectories onto a map designed around the vehicle position obtained by Global Positioning System (GPS) [8]. The trajectory-based methods show good performance where there is sufficient knowledge of the environment, but have difficulty in estimating the pedestrian trajectories accurately using only the images recorded during driving. 

The aim of the second approach tries to estimate the pedestrian intention by extracting some feature data, including pedestrian position, direction and magnitude of pedestrian movement, and pedestrian head pose. Huang et al. were the first to propose the pedestrian intention prediction system using head pose and pose change features [9]. They compared the performance using only the head pose feature with using multiple features including pedestrian position, velocity, and acceleration with head pose to show the importance of the head pose feature in predicting pedestrians’ intention. Keller et al. used a stereo camera to detect pedestrians and analyze their position and velocity. They also detected pedestrian facial images and analyzed the turn-rate of faces to predict their path [10]. In other research, Keller et al. proposed an algorithm to categorize the pedestrian’s behaviors into four actions: walking toward the curbside, walking lateral to the vehicle driving direction, stopping, and continuing walking [11]. Schneider and Gavrila developed a system that predicts whether a pedestrian will come near the vehicle or stop at a safe distance by building a Dynamic Bayesian Network (DBN) [12]. Kwak et al. proposed a pedestrian intention prediction algorithm based on Dynamic Fuzzy Automata (DFA), with a thermal camera for night usage [13]. They defined four states and determined the final intention among them using spatio-temporal features such as the distance between curbs and the pedestrian, the velocity of pedestrian, and pedestrian head orientation. 

The third approach, based on a skeleton estimation method, is similar to the first approach in that it detects a kind of pose, but different in that information about joint motion, especially knee motion, is the most important among the features used. Quintero et al. proposed several methods including Radial Basis Function-Support Vector Machines (RBF-SVM) [14], Gaussian Process Dynamical Models (GPDM) with Hidden Markov Model (HMM) [15], and Balanced-Gaussian Process Dynamical Models (B-GPDM) [16] to analyze pedestrian poses and classify them into several actions. 

Vision-based pedestrian detection and warning systems have been commercialized and installed on passenger cars since 2006, but the commercialized systems use only information about the distance between pedestrians and the vehicle. To overcome such a limit and improve the pedestrian safety system, many studies on predicting pedestrian intentions have been conducted, using various approaches. However, most studies on pedestrian intention seemed to be focused on categorizing the behaviors into a few types. The results may be important for a generic technology that could be utilized in many systems, but they do not seem to show how such categorized behaviors affect pedestrian safety. It also seems that the prediction of pedestrian intentions has not yet been considered in relation to protecting pedestrians. 

In this paper, we propose a vision-based pedestrian risk level estimation system. The proposed system uses distance information between pedestrians and the vehicle, like conventional commercialized products, but also makes additional use of relative position depending on the vehicle speed and movement information. The proposed system does not predict the pedestrian intention directly like other aforementioned methods, but we think that the pedestrian’s movement information reveals their intention. This is fully reflected in the fuzzy rule base inside the fuzzy system used to estimate their risk level. Formulating the prediction of the risk level using several factors based on the knowledge and experience of human drivers, especially pedestrian movement, allows the fuzzy system to make decisions that seem human-like. 

Following this introduction, Section 2 shows the overall system implementation. The input and output of the system are mentioned, and the information used inside the system is outlined. The hardware equipment used to obtain images and the vehicle speed are also introduced in Section 2. Section 3 explains the details of how the necessary information is extracted from the raw input data. Section 4 explains how fuzzy logic is used and how the rule base used for determining the risk level is designed. Actually, Section 3 and Section 4 give a detailed description of the two main software modules of the system described in Section 2. Section 5 describes the experimental setup, especially the test database used to validate the performance of the proposed system. Also shown here are the experimental results, from the viewpoint of how correctly the system evaluates the risk to pedestrians. Finally, Section 6 summarizes the work and the main contribution of this paper, and suggests future work for improving the performance of the proposed system.

## 2. System Overview

The system proposed in this paper estimates the odds that the ego-vehicle in motion will hit pedestrians who are detected by a camera installed on the windshield. This estimation of risk level is made by a fuzzy system, using pedestrian information that includes location and movement. The inputs for the proposed system are images captured by an on-dash camera in the car, and the vehicle speed measured by an external GPS module. The output of the system is a risk level designated by a number between 0 and 10. The higher the number is, the higher the risk of danger.

**Figure 1:**
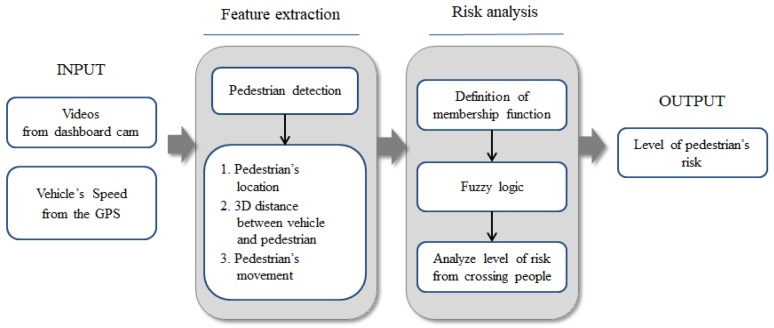
Overview of the proposed system: The system has two main modules, a feature extraction module and a risk analysis module. These take videos from a car dash camera and vehicle speed information from an external GPS module, and output the level of risk to pedestrians.

Internally, this system consists of two main software modules: a feature extraction module for extracting pedestrian information, and a risk analysis module for determining the risk level. The feature extraction module detects the pedestrians, if any, from images, and calculates their relative positions from the vehicle, as well as the stopping distance at the current speed of the vehicle. It then extracts the pedestrian movement information. Using all the information processed by the feature extraction module, the risk analysis module calculates the degree of risk using the fuzzy system. Finally, the system indicates the detection of a pedestrian with a bounding box around him/her, and overlays the risk level above the box on the original image. This system can be used as a driver assistance system such that, if the risk level exceeds a certain value, a warning message can be transferred in various ways. Figure 1 illustrates an overall overview of the proposed system. The details of the feature extraction, which is done using several computer vision techniques and risk analysis by the fuzzy system, will be explained in Section 3 and Section 4, respectively.

**Figure 2:**
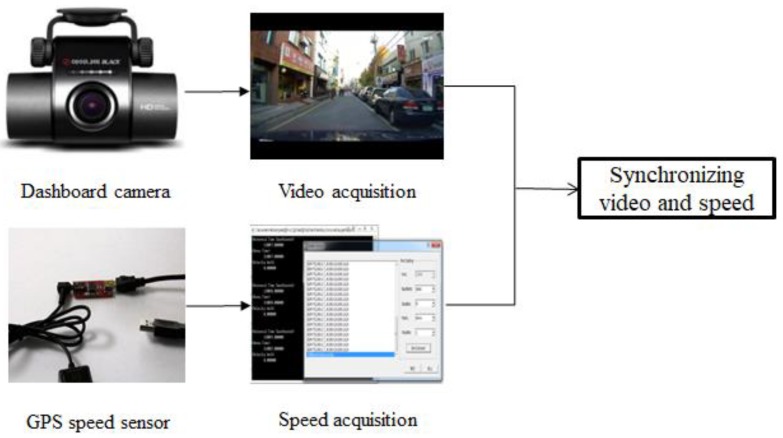
The speed information obtained from an external GPS module is written to a text file with the PC clock time. The speed information is synchronized with the video input from the on-dash camera in a car using the PC clock time afterwards.

The input images were obtained from a car dash CCD camera (BLACK-CLAIR, Inavi, Inc, Seongnam, Korea). This camera has a 146° field of view and produces 1280 × 720 pixel images recorded at 15 frames to 30 frames per second. However, for faster computation, the saved images were reduced to 320 × 240 pixel images before being used as input for the system. The vehicle’s speed information was obtained from a stand-alone GPS module (model AD-GPSSUIT V1.0, NewTC, Seoul, Korea). The speed information was updated once a second and written to a text file that was synchronized with the recorded images. The hardware setup used to obtain the input data is shown in Figure 2.

## 3. Extraction of Pedestrian Information

### 3.1. Position of Pedestrians

**Figure 3:**
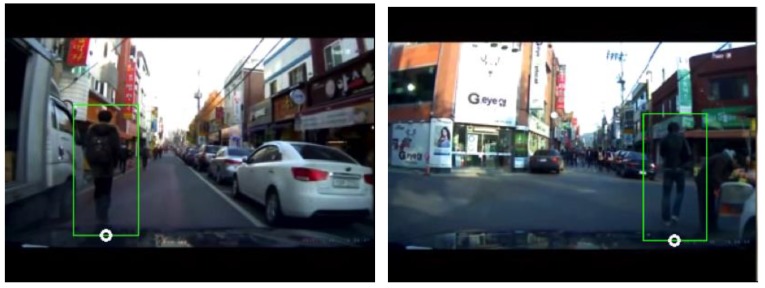
Detection of pedestrian using the histogram of gradient (HOG) + support vector machine (SVM) method. The pedestrian detected is marked with a green box bounding the pedestrian. The midpoint of the base of this box is used as the pedestrian’s position in the image.

The most prominent feature of the system proposed in this paper is the use of pedestrian relative position and movement, as well as the distance from the vehicle to the pedestrian, as the basic information for determining the collision risk level. To detect the pedestrians in the recorded images is the first and most important step in extracting valuable information. We applied a histogram of gradient (HOG) method [17] and linear support vector machine (SVM) [18], which have both been widely used for detecting objects using computer vision, and the related classes and functions are provided by OpenCV library. Figure 3 shows an example of a pedestrian detection result in which a bounded green box indicates the pedestrian detected. The position of the pedestrian is defined as the midpoint of the base of the green box, which is marked by small white circles in Figure 3.

### 3.2. Distance of Pedestrians

**Figure 4:**
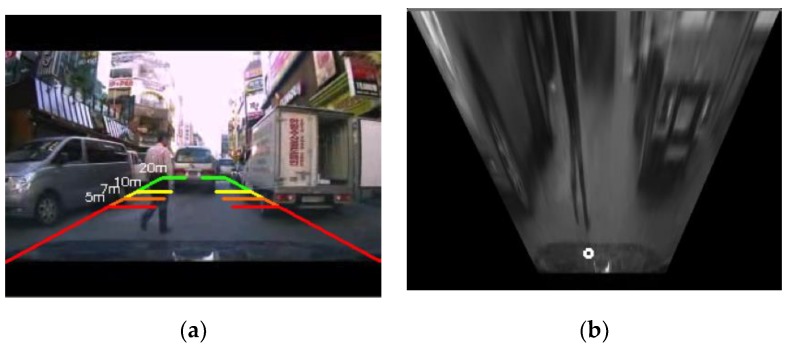
Transformation of the image from 2D to 3D. (**a**) Perspective 2D original image, (**b**) transformed 3D image: Because the distance should be computed in 3D view or top-view, the original perspective-view image is transformed using the homography transformation. Several distances (5 m, 7 m, 10 m, and 20 m) calculated in the transformed image are indicated on the original image, as shown in (**a**).

In order to obtain an accurate distance between a pedestrian and the vehicle, the perspective bird’s-eye view image from the in-dash camera should be transformed into a top-view image. This was done by homography transformation, by which the pixel coordinates in the image frame, $\left(\hat{X},\hat{Y}\right)$ are transformed into a 3D global coordinate, $\left(\hat{x},\hat{y}\right)$ through the following transformation

(1) $$\left[\begin{array}{c} \hat{x}\\ \hat{y}\\ 1 \end{array}\right]=\left[\begin{array}{ccc} H_{11} & H_{12} & H_{13}\\ H_{21} & H_{22} & H_{23}\\ H_{31} & H_{32} & H_{33} \end{array}\right]\left[\begin{array}{c} \hat{X}\\ \hat{Y}\\ 1 \end{array}\right]$$

where $H_{ij}$s are determined from the four independent matching points in the original frame and the transformed frame [19]. Figure 4 shows an example of an image transformed by homography transformation. In the transformed image, the distance of a pedestrian from the vehicle is estimated from the following equation.

(2) $$\Delta s=\sqrt{{\left(I_{px}-I_{vx}\right)}^{2}+{\left(I_{py}-I_{vy}\right)}^{2}}$$

where $\left(I_{px},I_{py}\right)$ is the coordinate of the pedestrian and $\left(I_{vx},I_{vy}\right)$ is the fixed coordinate of the center of the vehicle, both of which are in the transformed image.

### 3.3. Pedestrian Movement

Compared with the conventional methods that determine the safety level based mainly on the static position of the detected pedestrian, we also took into account the movement of the pedestrian. The advantage of this consideration seems clear when two situations are compared. In one case, a pedestrian on the left sidewalk is moving to the front of the vehicle and in the other, a pedestrian in the same position is just standing, or moving outside of view. It is clear that the former is more dangerous than the latter. Another situation to be considered is the comparison of pedestrians running toward the vehicle with those who are walking in the same direction that the car is traveling. In this case, the former is more likely to be in danger than the latter. 

This information about pedestrian movement was obtained from the optical flow, which denotes the movement of each pixel of an object or from a camera displacement with a vector field. The optical flow is based on three assumptions: brightness constancy, temporal persistence, and spatial coherence [20]. The original Lucas–Kanade method used a small local window around the interesting pixels when it tried to find matching pixels between the frames, so it could miss a large movement (that is, greater than the window size). The modified Lucas–Kanade method, which tracks the pixels from upper layer to lower layer of the pyramid images constructed from several images, can overcome this problem and detect a large movement [21], which is why it was used in this paper to obtain pedestrian movement information. 

Because the vehicle moves as well as pedestrians, the optical flow has information on the movement caused by the vehicle as well as that of pedestrians. Thus, it is necessary to distinguish, within the optical flow, the movement of pedestrian from that of the vehicle. This necessitates analysis of the optical flows in the overall image area. However, such analysis of the overall image area requires a large computational load, which means that it is difficult to process the algorithm in real-time. Therefore, in this paper, we reduced the area of interest to the bounding boxes, including the detected pedestrians, in the analysis of the optical flows.

Because many optical flow vectors exist inside a box, one optical flow vector was chosen to represent one box or, equivalently, one pedestrian. In this paper, the arithmetic average vector of all optical flow vectors inside the box was chosen to represent the pedestrian’s movement. That is, if there was n optical flow vectors with components $x_{i}$ and $y_{i}$ where i=1, 2,…, n in a box, the movement vector for the box would be $\left(v_{x},v_{y}\right)=\left(\sum_{i=1}^{n}x_{i}/n,\sum_{i=1}^{n}y_{i}/n\right)$. The direction of the pedestrian’s movement is then defined as $\mathrm{atan}2\left(v_{x},v_{y}\right)$, and the magnitude of the pedestrian’s movement is defined as $\sqrt{v_{x}{}^{2}+v_{y}{}^{2}}$.

## 4. Determining the Risk Level Using a Fuzzy System

**Figure 5:**
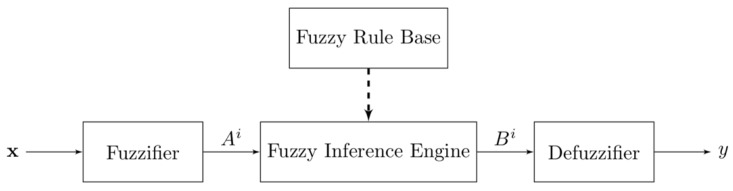
Basic configuration of fuzzy logic systems [22].

A fuzzy logic system can be defined as a nonlinear mapping from a real-valued input vector to a real-valued output value, or a knowledge-based system constructed from human knowledge in the form of fuzzy IF–THEN rules. The main blocks of a fuzzy logic system are fuzzifier, fuzzy rule base, fuzzy inference engine, and defuzzifier [22], as shown in Figure 5 where $x\;\in U\subset R^{n}$ is a real-valued input, y∈V⊂R is a real-valued output, $A^{i}$ is a fuzzy set in input space U, and $B^{i}$ is a fuzzy set in output space V [22]. A fuzzy set is a set of pairings of elements of the universe of discourse, coupled with their associated membership values between 0 and 1, which are determined by membership functions. 

The fuzzy logic works as follows: First, the fuzzifier transforms a real-valued variable into a fuzzy set using user-defined fuzzy linguistic variables, terms, and membership functions. The linguistic variables are the input and output variables, the values of which are words from a human language, instead of numerical values. Depending on the converted fuzzy sets, the fuzzy IF–THEN rules are then evaluated and their results are combined by fuzzy inference engine and fuzzy rule base. The IF–THEN rules are also represented in statements from a human language. Lastly, the resulting fuzzy output is transformed into a crisp, real-valued output using the membership functions by the defuzzifier. The detailed mathematical description of the fuzzy logic system can be found in any reference or textbook on fuzzy system, e.g., Ref. [22]. 

The fuzzy system constructed in this paper had four fuzzy inputs and one fuzzy output. The details of the fuzzy inputs and output are listed in Table 1. The labels for the MovDirection fuzzy input variables, NE, NW, SW, and SE, denote the northeast (up and right), northwest (up and left), southwest (down and left), and southeast (down and right) directions in the virtual image coordinate, respectively. Except for these labels, most labels for the other fuzzy sets are self-explanatory.

**Figure 6:**
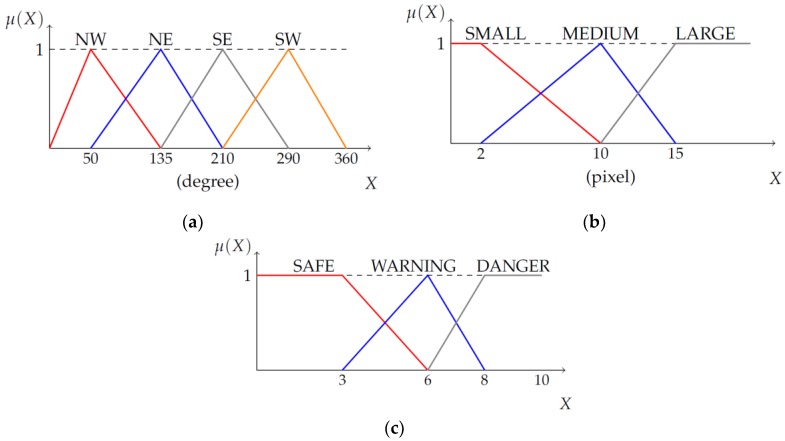
Membership function for two fuzzy inputs and a fuzzy output. (**a**) MovDirection, (**b**) MovMag, (**c**) Result.

The membership functions for MovDirection, MovMag, and Result are shown in Figure 6, and those for the other fuzzy variables, PedPos and PedDist, will be explained in detail in the following subsections.

### 4.1. Membership Functions for Pedestrian’s Position

**Figure 7:**
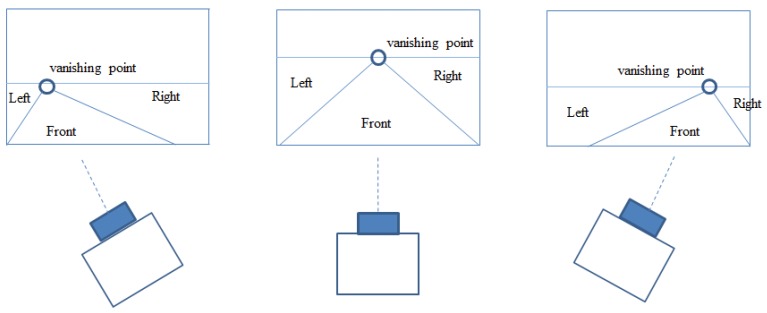
Classification of pedestrian’s location based on the vanishing point and two lines: The vanishing point and two lines move accordingly when the vehicle turns left or right.

The position of a pedestrian, which was obtained by the feature extraction module explained in Section 3, was fuzzified into three fuzzy variables (LEFT, FRONT, and RIGHT) by input membership functions. However, the same image point can belong to a different area depending on the vehicle’s heading (direction), as illustrated in Figure 7. Therefore, the shape of the membership functions should change accordingly, which was done by finding left and right lines in the image.

**Figure 8:**
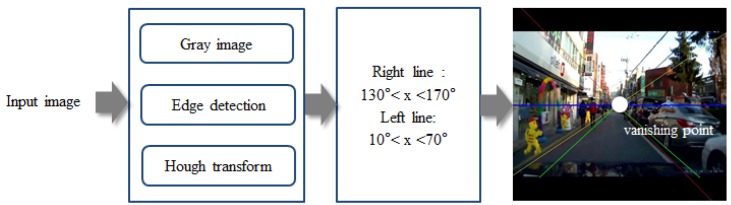
Steps for finding the two lines that will be used as guidelines for classification of the pedestrian’s location: The right panel shows an example of this process where more than two lines are detected from a line detection algorithm, the Hough transform. Among those lines, two lines are selected between specific angles.

The two lines used for shaping the membership functions were detected by Sobel edge extraction and the Hough transform. Among the several lines detected, one for which the slope was between 10° and 70° and another for which the slope was between 130° and 170° were selected. The steps and an example for finding the two lines and a vanishing point in an image are illustrated in Figure 8.

**Figure 9:**
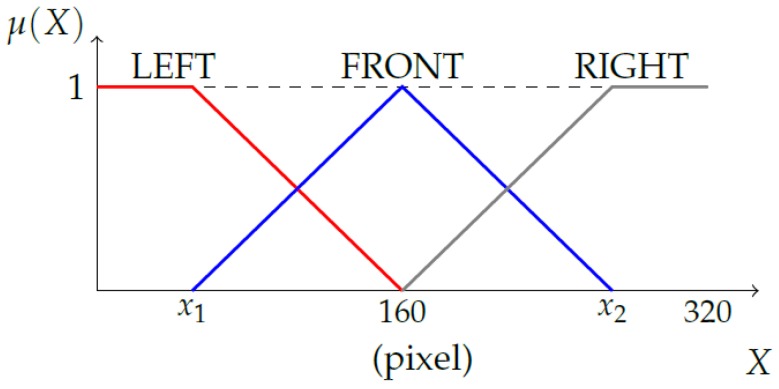
Membership function for the pedestrian’s position (PedPos): The values of $x_{1}$ and $x_{2}$ are determined by the *x*-coordinate of the two lines in the transformed 3D image. The two lines are detected by the process explained in Figure 8.

Let the *x*-coordinates of the two lines in the 3D image transformed by the homography transformation explained in Section 3.2 be $x_{1}$ and $x_{2}$. The membership functions for the pedestrian position are then chosen as shown in Figure 9.

### 4.2. Membership Function for Pedestrian’s Distance

**Figure 10:**
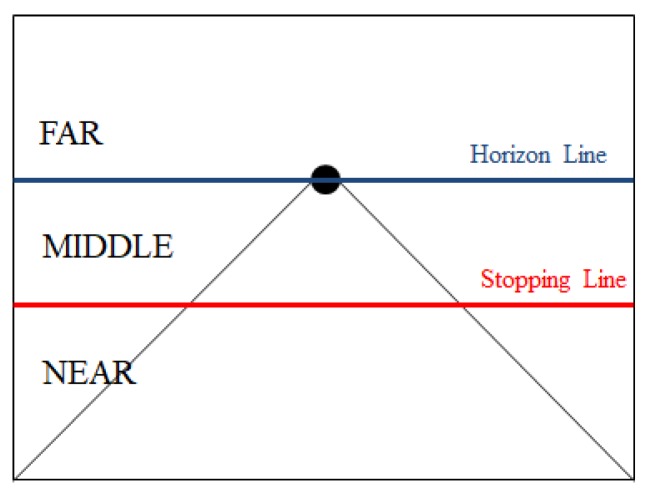
Conceptual classification of the pedestrian’s distance: The distance between a pedestrian and the ego-vehicle is classified as one of three regions, determined by the horizontal line passing through the vanishing point and the stopping distance line.

The pedestrian distance was fuzzified into three fuzzy variables (NEAR, MEDIUM, and FAR) by input membership functions. Considering that the probability of collision is related to both how far the pedestrian is located from the car, and to how long the vehicle takes before it can fully stop, the shape of membership functions for distance was designed to change with the vehicle’s stopping distance. This idea is illustrated in Figure 10.

The stopping distance consists of reaction distance and braking distance, where the reaction distance refers to the distance a vehicle travels from the point when a driver recognizes he should apply the brake to the point when he actually applies the brake. The braking distance refers to the distance a vehicle will travel from the point when its brakes are fully applied to the point when it comes to a complete stop. The reaction distance (m), $d_{1}$ is calculated by $d_{1}=\left(vehicle\;speed\right)\left(m/s\right)\times \left(reaction\;time\right)\;\left(s\right)$ where (reaction time) is about ~0.7–2.5 (s); National Highway Traffic Safety Administration (NHTSA) uses 1.5 s for the average reaction time [23]. From Newton’s second law and the friction force equation, the braking distance (m), $d_{2}$ is calculated by

(3) $$d_{2}=\frac{v^{2}}{2\mu g}$$

where *v* is the vehicle’s speed (m/s), μ is the coefficient of friction, and g is the gravitational acceleration, 9.8 ($m/s^{2}$). In this paper, μ was 0.8. Because the vehicle speed was obtained from a GPS receiver once a second, the stopping distance, $d_{1}+d_{2}$, was calculated once per second.

**Figure 11:**
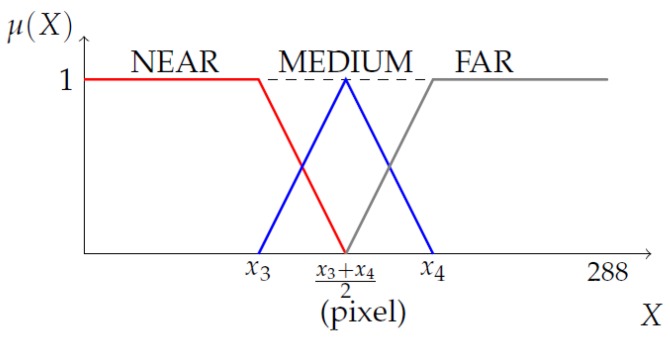
Membership functions for pedestrian’s distance (PedDist): The value of $x_{3}$ and $x_{4}$ are determined by the x-coordinate of two lines in the transformed 3D image.

Let the distance corresponding to the stopping distance and the horizontal line passing through the vanishing point be $x_{3}$ and $x_{4}$ in the transformed 3D image, respectively. The membership functions for the pedestrian distance were chosen as shown in Figure 11.

### 4.3. A Fuzzy Rule Base

The heart of a fuzzy system is a collection of so-called fuzzy IF–THEN rules. In our case, an example of IF–THEN rules is

- IF PedPos is “LEFT” and PedDist is “MEDIUM” and MovDirection is “NW” and MovMag is “SMALL”, THEN Result is “SAFE”.

The above rule was made following the usual judgement such that, if when a driver detects a man on the left side of vehicle and he is a little away and moving to the driver’s left side, that is, moving away further from the vehicle, then the driver thinks he is quite safe from a crash. Because the total possible combination of four inputs with their membership functions is 3×3×4×3=108; the total possible number of rules in the fuzzy rule base is 108. Among them, we combined some rules into simpler ones, and ended up with 64 fuzzy rules. Two examples in the final rule base are

- IF PedDist is “FAR”, THEN Result is “SAFE”
- IF PedPos is “FRONT” and PedDist is “NEAR”, THEN Result is “DANGER”.

The first rule above means that, if the pedestrian locates quite far away, he is safe for now independent of any other factors. Similarly, the second one above means that, if the pedestrian locates very near in front of the vehicle, he is absolutely in danger, without need to consider any other information. The fuzzy reasoning follows the Mamdani reasoning method with max–min operation, and defuzzification was done by the center of gravity method [22]. The defuzzified crisp value lies between 0 and 10, which is the output of the proposed system.

## 5. Experimental Results

### 5.1. Experimental Setup

The proposed system was implemented using C++ with OpenCV 2.3 and run on a PC with an Intel i7 quad-core CPU and 6 GB RAM. Under this PC environment, the average processing speed of the proposed system was measured to be 21 frames per second (fps). The test images were recorded by ourselves with a car dash camera installed above 120 cm from the ground. The videos were recorded in the daytime at several vehicle speeds, from 0 to 30 km/h. Because the main target of the new system was to detect pedestrians and to predict the risk of collision, the recordings were made on small streets where pedestrians and vehicles mixed closely (e.g., densely concentrated shopping areas around an apartment complex or a university campus). The video database contents were grouped into eight sets. Table 2 lists the eight groups with their group names, the number of frames in the group, and shooting locations.

### 5.2. Performance Analysis

**Figure 12:**
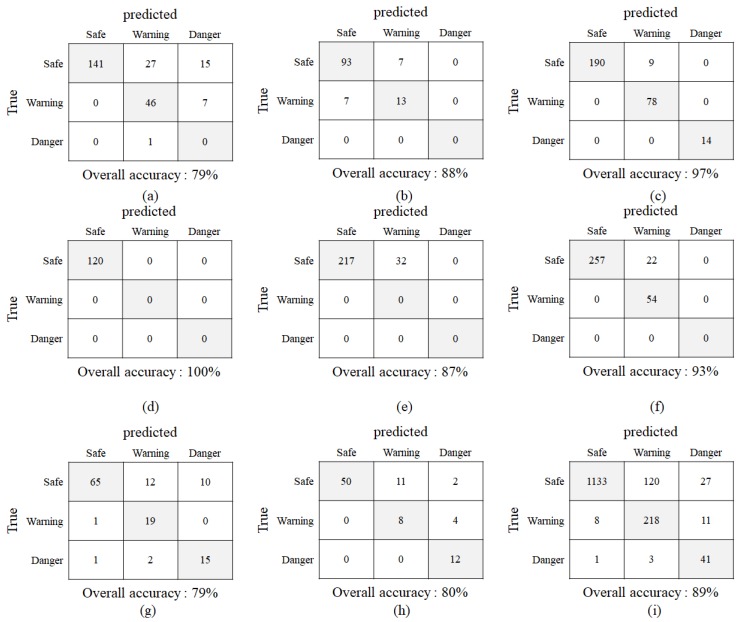
Confusion matrices for each dataset and total database. (**a**) ATP_S1 dataset, (**b**) ATP_S2 dataset, (**c**) ATP_S3 dataset, (**d**) ATP_S4 dataset, (**e**) ATP_S5 dataset, (**f**) STORE_S1 dataset, (**g**) UNIV_S1 dataset, (**h**) UNIV_S2 dataset, (**i**) Total: The columns of the matrix denote ground truths made by humans, and the rows of the matrix denote decisions made by the proposed system. All correct predictions are located in the diagonal of the table.

To evaluate the performance of the proposed system, we generated confusion matrices for each dataset. The confusion matrix is a specific tabular layout that is often used to describe the performance of a classifier on a set of test data for which the true values are known [24]. Each row of the confusion matrix represents the ground truth, and each column represents the classification results of the system. The ground truth was made manually by researchers for each bounding box including a pedestrian, and the classification results were obtained from the output of the proposed system. The bounding boxes with risk levels of 0–5, 5–8, and 8–10 were labeled as SAFE, WARNING, and DANGER, respectively. The confusion matrices for each dataset and total data are shown in Figure 12. Based on the confusion matrices, we calculated the classification recognition rate using the following equation. The performance results show a 79%–100% recognition rate in the eight datasets, and 89% in total.

(4) $$\mathrm{accuracy}=\frac{\mathrm{the}\;\mathrm{number}\;\mathrm{of}\;\mathrm{correct}\;\mathrm{recognized}\;\mathrm{samples}}{\mathrm{the}\;\mathrm{number}\;\mathrm{of}\;\mathrm{total}\;\mathrm{samples}}\times 100\left(\%\right)$$

**Figure 13:**
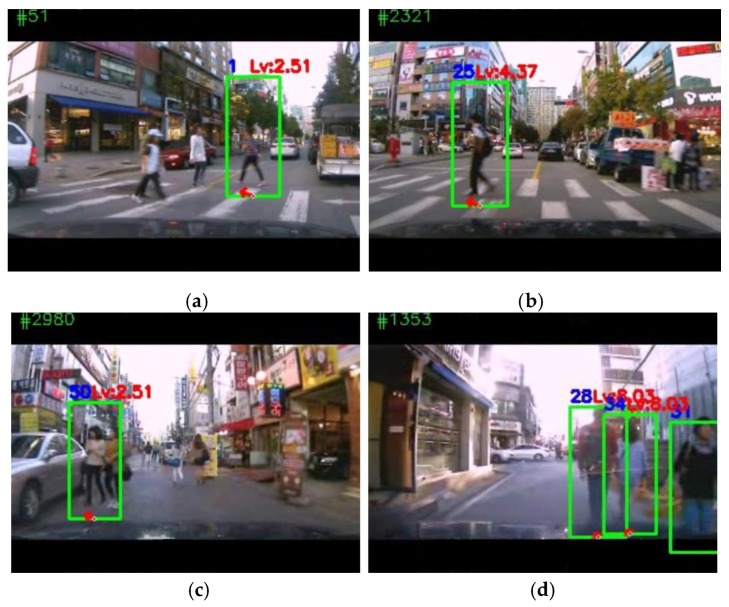
Some working examples of the proposed system. (**a**) Frame 51 of the APT_S1 dataset, (**b**) frame 2321 of the APT_S1 dataset, (**c**) frame 2980 of the UNIV_S1 dataset, (**d**) frame 3273 of the UNIV_S1 dataset. The results are illustrated with green bounding boxes around the detected pedestrians, the red arrows indicate the direction of pedestrian movement, and the red risk level, above the green box, was obtained from the fuzzy system. Along with the information around the boxes, the frame number and vehicle speed, as well as the calculated stopping distance at that time, are displayed on the top border above the image. The estimated pedestrian position, distance from the vehicle, and the direction of movement are displayed on the border below the image.

More detailed analysis of the experimental results shows the validity and vulnerability of the proposed system’s performance. Figure 13 illustrates some examples of results from a few of the different datasets. In Figure 13a (frame number 51), it can be seen that a pedestrian was detected 81.54 pixels away in the front of a vehicle that was turning right, and was moving to the left. In Figure 13b, a pedestrian was also detected in front of the vehicle, which is going straight, while the pedestrian was going to the left. The two pedestrians were located in the same relative position (in front of the vehicle) and moving in the same direction (left). However, because the pedestrian in the Figure 13a was farther away than the one in Figure 13b, and because the vehicle speed in the frame with Figure 13b was higher than of Figure 13a, the stopping distance was longer for Figure 13b than for Figure 13a. Therefore, the level of risk to the pedestrian in Figure 13b (4.37) was higher than to the pedestrian in Figure 13a (2.51), which makes sense. The pedestrian in the frame of Figure 13c was detected near the vehicle (distance calculated was 47.63 pixels), but was to the left of the vehicle and moving left. Thus, her risk level was judged similar to that for Figure 13a and lower than for Figure 13b, which appears to be a correct judgement. As shown in Figure 13d, the proposed system can detect one or more than one pedestrians and evaluate their risk levels simultaneously.

**Figure 14:**
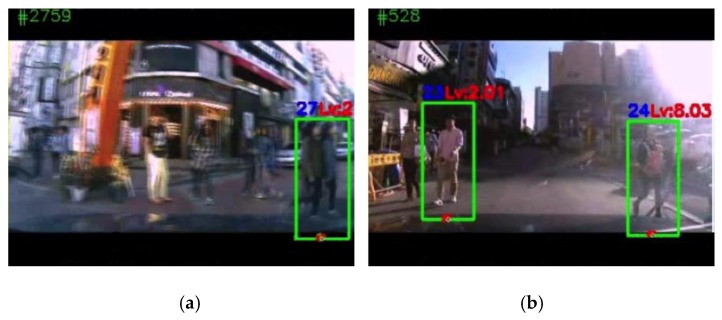
Two examples of failure of the proposed system: (**a**) Frame 2759 of the APT_S1 dataset. Due to the blurred image, many pedestrians were not detected. (**b**) Frame 526 of the APT_S1 dataset. Incorrect feature extraction and direction of movement for the right pedestrian led to an incorrect judgement. That is, she was quite safe actually, but got a high risk level.

However, there exist frames for which the proposed system made wrong decisions. Analyzing the failures revealed two main factors. One was the failure to detect pedestrians due to image processing problems, such as blurs by any cause, such as that shown in Figure 14a. In such cases, many pedestrians in front of the vehicle were not detected at all. The other factor was incorrect information extraction due to external disturbances such as the vehicle’s up and down motion, vehicle’s left/right turn, or abrupt change in illumination, as shown in Figure 14b. The right detected pedestrian in Figure 14b is moving to the right, but the moving direction was calculated to be to the left by the system. In addition to wrong information for the movement direction, because the pedestrian distance was calculated to be very short, the system produced a very high risk level (8.03). However, in this case, the pedestrian was determined to be safe by a human observer.

## 6. Conclusions

In this paper, we proposed a system that estimates the risk level of pedestrians in front of driving vehicles using fuzzy logic. The inputs included the pedestrian position, relative distance from the vehicle, vehicle speed, and the direction and magnitude of the pedestrian movement. This system requires only a car dash camera, which is widely used in many vehicles, and a low-priced GPS receiver for collecting raw data. Compared with conventional methods that give warning whenever any pedestrian is detected in front of the vehicle, the proposed system determines different risk levels for each pedestrian detected in the camera image. The system also takes their motion and the vehicle speed into account when it calculates the risk level. In order to analyze the performance of the proposed system, we built a test database including eight different datasets recorded by ourselves on narrow streets where pedestrians and vehicles mixed closely. With this database, the proposed system showed an 89% recognition rate in total. Analysis of the failure cases showed that the system was weak when the vehicles turned left or right, or passed a speed bump. To overcome these shortcomings, it seems necessary to adopt another sensor and to apply a kind of sensor fusion method. The proposed system uses fuzzy logic to determine the risk level from inputs, but it would be possible to use a kind of machine learning algorithm (one of the popular topics in computer vision), and to compare the characteristics of the two systems. For this approach, it would be important to build a database large enough to train the learning algorithm, which requires much time and effort for even large research groups.

## Figures and Tables

**Table 1:** Fuzzy variables and fuzzy sets.

Input/Output	Meaning	Fuzzy Variables	Fuzzy Sets
Input	Pedestrian’s position	PedPos	LEFT, FRONT, RIGHT
	Pedestrian’s distance	PedDist	NEAR, MIDDLE, FAR
	Direction of pedestrian’s movement	MovDirection	NE, NW, SW, SE
	Magnitude of pedestrian’s movement	MovMag	SMALL, MEDIUM, LARGE
Output	Risk level	Result	SAFE, WARNING, DANGER

**Table 2:** The test video clips collected.

Video Group	No. of Frames	Scene Description
APT_S1	5566	shopping area around an apartment complex
APT_S2	6939	shopping area around an apartment complex
APT_S3	6211	shopping area around an apartment complex
APT_S4	1863	shopping area around an apartment complex
APT_S5	4306	shopping area around an apartment complex
STORE_S1	5677	streets in front of a department store
UNIV_S1	6184	streets in front of a university campus
UNIV_S2	3124	streets in front of a university campus
